# Effects of Dental Rehabilitation under General Anesthesia on Children's Oral Health-Related Quality of Life Using Proxy Short Versions of OHRQoL Instruments

**DOI:** 10.1155/2014/308439

**Published:** 2014-01-23

**Authors:** Ziad D. Baghdadi

**Affiliations:** ^1^Department of Preventive Dentistry, Riyadh Colleges of Dentistry and Pharmacy, P.O. Box 67126, Riyadh 11596, Saudi Arabia; ^2^Department of Community Health and Epidemiology Saskatchewan University College of Medicine, Saskatoon, SK, Canada S7N 5E5

## Abstract

*Aim*. To examine the impact of comprehensive dental treatment under general anesthesia (GA) on oral health-related quality of life (OHRQoL) in children using short form versions of the Parental-Caregivers Perceptions questionnaire (P-CPQ) and Family Impact Scale (FIS). *Design*. A pretest/posttest study involved parents whose children (*N* = 67) were affected with severe childhood caries and completed comprehensive dental treatment under GA. All parents completed the short form versions of the P-CPQ and FIS at baseline and 4–8 weeks following the dental treatment. To examine test-retest reliability, a convenience sample of 38 parents repeated the pretreatment questionnaires 1-2 weeks after they completed them at baseline. Statistical tests including the Kruskal-Wallis test, Cronbach's alpha, and paired *t*-test were used to examine cross-sectional construct validity, internal consistency, and responsiveness of the instruments, respectively. *Results*. Cross-sectional construct validity and internal consistency were acceptable. Test-retest reliability was excellent. Large decreases in posttreatment scores were observed along with moderate to large effect sizes. *Conclusions*. Dental treatment under GA is associated with considerable improvement in OHRQoL of children and their families, as demonstrated by short form versions of the P-CPQ and FIS completed by the children's parents.

## 1. Introduction

There has been a recent interest in measuring how oral disease and subsequent treatment affect an individual's quality of life. Several questionnaires measuring oral health-related quality of life (OHRQoL) have been developed and validated in several languages and used for public health policies, research, and clinical practice [1]. Using a scoring system, OHRQoL questionnaires gauged children's and adolescents' responses to therapeutic interventions of oral conditions, including caries treatment, traumatic dental injuries treatment, and orthodontic treatment [1]. There are two systematic reviews that evaluate the quality of the evidence about changes in children's OHRQoL following dental treatment [2, 3]. The most recent systematic review selected and analyzed 9 articles (out of 1,044 initially retrieved), which met the inclusion criteria [2]. Of these, Taylor et al. [4] found that malocclusion and its treatment do not appear to affect OHRQoL. Jabarifar et al. [5] and Agou et al. [6] found that dental treatment of caries and orthodontic treatment had a significant effect on OHRQoL, but without significant reduction in scores. Malden et al. [7] and Klaassen et al. [8] found significant reductions of impact scores after treatment of caries in children. Similar reductions in scores were reported by Berger et al. [9] after treatment of severe dental injuries. Li et al. [10] found that the OHRQoL instrument had some limited ability to respond to change.

OHRQoL questionnaires, including Child Perception Questionnaire (CPQ11-14), the Parent-Caregivers Perceptions Questionnaire (P-CPQ), and the Family Impact Scale (FIS), were translated into Arabic and validated in Saudi Arabian samples [11, 12]. To date, all published studies report how oral disease affects life quality of patients in different settings; however, there is a lack of studies on how dental interventions impact OHRQoL. Dawoodbhoy et al. [13] using the Arabic version of the CPQ11-14 found that only very severe malocclusion (i.e., handicapping) had an impact on the quality of life of participating children. In an earlier study, Brown and Al-Khayal [11] found PCPQ11-14 valid and reliable for use in Saudi Arabia, but they recommended development of a shorter version of the questionnaire. On a similar note, the need of short form versions was signaled by Jokovic et al. [14], who thought that routine use of OHRQoL questionnaires could be limited by their length concomitant with respondent burden. Thomson et al. [15] using a sample of young children from New Zealand developed short form versions of the P-CPQ and FIS. The purpose of this study was, therefore, to use the short form versions of the P-CPQ and FIS to examine the impact of comprehensive dental treatment under general anesthesia on the life quality of the children and families. As Thomson et al. [15] failed to examine test-retest reliability of the short form versions, an additional purpose was to examine test-retest reliability of the short form questionnaires.

## 2. Materials and Methods

The protocol was cleared by the Research Center of Riyadh Colleges of Dentistry and Pharmacy and written informed consents were obtained from all patients' parents/caregivers. The sample size was determined based on the lowest effect size (0.28) reported by Gaynor and Thomson [16] on the family conflict domain of the FIS. With 80% power and 0.05 alpha, it was determined that 67 cases would be needed. For testing reliability, it was determined that 39 subjects would be needed for 80% probability difference and 20% error margin.

The patients were recruited consecutively and included all children (age ranges 3–10 years) requiring comprehensive dental treatment under general anesthesia. The pediatric dental patients were referred to be treated under general anesthesia when nonpharmacological behavior guidance techniques were not viable (e.g., acute situational anxiety, uncooperative behavior, and invasive procedures such as multiple extractions that are psychologically threatening the patient). To be eligible for the study, the patient should be classified as ASA I or ASA II; patients with compromised general health due to serious ailments or disabilities were excluded. Child patients with special health care needs that may affect their quality of life were also excluded.

The short form version of the OHRQoL questionnaire has two main sections: the P-CPQ and the FIS. The P-CPQ consists of 16 items (known as 16-item P-CPQ), which are divided into 4 domains (or subscales): oral symptoms (OS, 4 items), functional limitations (FL, 4 items), emotional wellbeing (EWB, 2 items), and social wellbeing (SWB, 6 items). The FIS consists of 8 items (known as 8-item FIS), which are divided into 3 domains: family activity (FA, 4 items), parental emotions (PE, 2 items), and family conflict (FC, 2 items). The total number of items is 24, comparing to 49, the total number of items of the full form questionnaire [15, 16]. All items sought information on the frequency of impacts and, therefore, were scored using a 4-point Likert scale (never, 0; once or twice, 1; sometimes, 2; often, 3, and every day or almost every day, 4). As either the father or the mother of the child was asked to complete the questionnaire, it was necessary to include a “Don't know” response to prevent loss of valuable information in case the respondent had no information answering a particular item. A score of 0 was given to such an item. A recent study [12] evaluating the OHRQoL of Saudi Arabian autistic children compared to their nonautistic siblings reported that some parents, fathers in particular, noted that they lack the knowledge to answer some items with certainty. It was thought that adding a “Don't know” response might solve this problem. The pretreatment OHRQoL questionnaires include a global transition item, “How much is your child's overall wellbeing affected by the condition of his/her teeth, lips, jaw, or mouth?” The posttreatment OHRQoL questionnaires include a global transition item, “Since the operation to fix his/her teeth, is your child's overall quality of life: Much improved, A little improved, The same, A little worse, or Much worse.” The children's parents completed the OHRQoL questionnaires twice: baseline which was at the treatment plan/case presentation session and follow-up which was 4–8 weeks after dental treatment. A convenience sample of parents was asked to complete another set of pretreatment questionnaires while their children undergoing GA, usually within 1-2 weeks after treatment plan sessions.

Full P-CPQ and FIS total scores were computed by summing scores for all 24 items. Subscales scores were obtained for P-CPQ and its domains and FIS and its domains by summing discrete subsets of items within the categories mentioned above. Cross-sectional construct validity was evaluated by examining the association between means of pretreatment scores and the rating of the pretreatment global transition item. The Kruskal-Wallis test was used to test the associations. Internal consistency reliability was examined using Cronbach's alpha. Test-retest reliability was examined using Interclass Correlation Coefficient (ICC). For examining responsiveness of the questionnaires, change scores were computed by subtracting posttreatment scores from pretreatment scores. A paired *t*-test was used to examine changes. Effect size (ES) statistics were also calculated by dividing the mean of change scores by the standard deviation (SD) of the pretreatment scores. ES gives a dimensionless measure of effect; ES less than 0.2 indicates a small clinically meaningful magnitude of change, 0.2–0.7 a moderate change and above 0.7 a large change. All tests were computed using SPSS version 20 with the 0.05 level of significance.

## 3. Results

Sixty-seven children completed dental treatment under GA, of whom 36 were boys with a mean age of 6.07 (SD 2.04) and 31 were girls with a mean age of 6.24 (SD 2.09). The parents who completed both pretreatment and posttreatment questionnaires were 25 fathers with a mean age of 42.6 (SD 9.81) and 42 mothers with a mean age of 39.1 (SD 7.8). Thirty-eight parents completed the pretreatment questionnaires a second time to examine test-retest reliability. All children received comprehensive dental treatment, including restorations, pulp therapy, stainless steel crowns (SSCs), and/or extractions, in addition to preventive procedures (dental prophylaxis, dental sealants, and topical fluoride). The mean number of restorations per patient was 6.82 (SD 2.87), pulp therapy 2.76 (SD 2.05), SSCs 0.80 (SD 1.35), and extractions 2.08 (SD 2.26). The mean dmft score was 9.94 (SD 3.24).

Data on the cross-sectional concurrent validity and Cronbach's alpha values of the P-CPQ and FIS and their domains are presented in Table 1. There were statistically significant gradients in mean scores consistent with the rates of response to the pretreatment global transition item. Those whose wellbeing was rated as more severely affected got higher scores, and vice versa. The only exception was in SWB where the mean score for those reporting “Some” was slightly lower than those reporting “A lot/Very much.” Cronbach's alpha values for the 16- and 8-item P-CPQ and FIS were 0.81 and 0.67, respectively, indicating an acceptable level of internal consistency. Test-retest reliability results are presented in Table 2. ICC values were 0.93 and 0.84 for 16-item P-CPQ and 8-item FIS, respectively, indicating excellent agreement with repeated administration of the questionnaires.

The mean pre- and posttreatment scores, the mean of change in scores, and effect sizes are presented in Table 3. The P-CPQ and the FIS scores showed significant decreases following dental treatment, along with large effect sizes. Only SWB and PE demonstrated moderate effect sizes. These results indicate that the scales are responsive to the positive changes brought about by dental treatment under GA. The minimally important difference (MID) is usually determined in such studies from the mean change in scores of those whose parents reported “A little” improvement when answering the posttreatment global transition item [16]. In the current study, only 2 parents reported “A little” improvement, in addition to 3 who reported no change (“The same”), and 62 who reported “Much improved.” The lack of variation in responses and the small number of cases in cells precluded the calculation of MID.

## 4. Discussion

The aim of this study was to evaluate the impact of dental treatment under GA on the quality of life of children affected by severe childhood caries using short form versions of the OHRQoL questionnaires. The results showed that the short form versions of the P-CPQ and the FIS could detect the positive changes resulting from comprehensive dental treatment, as perceived by the parents of child patients. In addition, both scales have adequate reliability and their responsiveness was acceptable in the sample used. These results are consistent with similar studies [5, 16] examining the impact of dental treatment under GA on children using full form versions of the P-CPQ and FIS. While the present study showed that the 16- and 8-item short form versions of the P-CPQ and FIS have an acceptable level of internal reliability, test-retest reliability, validity, and responsiveness, they cannot be recommended as definitive measures for use in clinical and epidemiological research before undertaking further studies. These studies should consider a wider range of oral and dental conditions. The current study involved children with severe childhood caries and, therefore, may or may not be valid or responsive in children with less severe caries or, say, having orthodontic problems. Other oral conditions that require further studies include, for example, orofacial clefts and periodontal diseases. The results of these proposed studies should confirm whether short form versions of OHRQoL questionnaires are condition-specific or generic. Comparing the sample of the current study to other studies [5, 7, 16] reveals that we used a relatively smaller sample. However, a post hoc power analysis using the smaller effect size obtained with the PE domain of the FIS (0.65) indicated that the required size of the follow-up sample with 0.80 power would be 29, which is considerably smaller than the 67 used here. As there are some conflicting results in the literature [17] when comparing between parents' and children's rating of OHRQoL, it is essential to conduct another study measuring self-reported OHRQoL in children. A shorter version of the P-CPQ (8-item) was recently developed by Thomson et al. [15] and needs further validation, exploring whether additional deletion of items would negatively affect face and content validity of the scales being used.

## 5. Conclusion

The results of this study reveal that the short form versions of the P-CPQ and FIS appear to be responsive to the positive changes associated with comprehensive dental treatment under GA, as reported by the parents of child patients. The instruments were also found to be valid for measuring OHRQoL in children affected by severe childhood caries.

## Figures and Tables

**Table 1:** Pretreatment Parental-Caregivers Perceptions Questionnaire (P-CPQ) scale (and subscale), Family Impact Scale (FIS), and Cronbach's alpha.

	How much is your child's overall wellbeing affected by the condition of his/her teeth, lips, jaws, or mouth?					
	Not at all	Very little	Some	A lot/very much	*P* value	Alpha
Number (%)	6 (9%)	12 (17.9%)	11 (16.4%)	38 (56.7%)	—	—
P-CPQ	8.16 (13.25)	14.75 (9.78)	20.36 (6.71)	33.39 (9.29)	0.008	0.81
P-CPQ domain						
OS	2.83 (4.07)	5.91 (4.1)	6.81 (2.08)	8.86 (3.03)	0.002	0.61
FL	3 (4.47)	3.91 (3.52)	5.81 (2.31)	6.71 (3.99)	0.035	0.61
EWB	1.16 (2.4)	1.83 (1.52)	3.18 (1.16)	3.6 (2.0)	0.004	0.49
SWB	1.16 (2.4)	3.08 (2.53)	4.54 (3.98)	3.21 (3.97)	0.20	0.66
FIS	4.83 (8.08)	7.83 (4.72)	11.18 (4.14)	12.28 (4.60)	0.004	0.67
P-CPQ + FIS	13 (21.13)	22.58 (13.82)	31.54 (7.67)	34.68 (11.22)	0.002	0.84

P-CPQ: Parental-Caregivers Perceptions Questionnaire; OS: oral symptoms; FL: functional limitations; EWB: emotional wellbeing; SWB: social wellbeing; FIS: Family Impact Scale; FA: family activity; PE: parental emotions; FC: family conflict.

**Table 2:** Mean overall and domain scores in the Parental-Caregivers Perceptions Questionnaire (P-CPQ) and Family Impact Scale (FIS) at baseline and follow-up, with effect sizes.

	OHRQoL mean scores (pretreatment/posttreatment) and effect size					
	Pretreatment (SD)	Posttreatment (SD)	Change in score (SD)	*P* value	Effect size (ES)	ES description
P-CPQ	19.41 (10.25)	2.80 (3.71)	16.61 (9.94)	<0.001	1.62	Large
OS	7.46 (3.66)	0.89 (1.08)	6.56 (3.64)	<0.001	1.79	Large
FL	5.73 (3.89)	1.0 (1.51)	4.73 (3.65)	<0.001	1.21	Large
EWB	3.22 (3.66)	0.67 (1.69)	2.76 (2.11)	<0.001	0.75	Large
SWB	3.22 (3.66)	0.67 (1.69)	2.55 (3.69)	<0.001	0.69	Moderate
FIS	10.64 (5.41)	2.59 (2.82)	8.04 (5.38)	<0.001	1.48	Large
FA	6.11 (3.30)	0.77 (1.26)	5.34 (3.30)	<0.001	1.61	Large
PE	2.47 (1.61)	1.41 (1.85)	1.05 (1.93)	<0.001	0.65	Moderate
FC	2.04 (1.91)	0.40 (0.88)	1.64 (1.94)	<0.001	0.85	Large

P-CPQ: Parental-Caregivers Perceptions Questionnaire; OS: oral symptoms; FL: functional limitations; EWB: emotional wellbeing; SWB: social wellbeing; FIS: Family Impact Scale; FA: family activity; PE: parental emotions; FC: family conflict; SD: standard deviation.

**Table 3:** Test-retest reliability measured for the total P-CPQ and FIS and for their domains: intraclass correlation coefficients (ICCs), item means, and item range.

	ICC	Item means	Range
P-CPQ	0.93	17.65	16.94–18.36
FIS	0.84	10.17	9.86–10.47
P-CPQ + FIS	0.94	27.82	26.81–28.84
OS	0.91	6.35	6.05–6.65
FL	0.93	5.34	5.05–5.63
EWB	0.76	2.61	2.57–2.65
SWB	0.86	3.34	3.26–3.42
FA	0.82	5.26	4.78–5.73
PE	0.69	2.65	2.55–2.76
FC	0.82	2.25	2.18–2.31

P-CPQ: Parental-Caregivers Perceptions Questionnaire; OS: oral symptoms; FL: functional limitations; EWB: emotional wellbeing; SWB: social wellbeing; FIS: Family Impact Scale; FA: family activity; PE: parental emotions; FC: family conflict.
